# Long-term cognitive performance and its relation to anti-inflammatory therapy in a cohort of survivors of severe COVID-19

**DOI:** 10.1016/j.bbih.2022.100513

**Published:** 2022-09-18

**Authors:** Harmke B. Duindam, Roy P.C. Kessels, Bram van den Borst, Peter Pickkers, Wilson F. Abdo

**Affiliations:** a Radboud University Medical Center, Department of Intensive Care Medicine, Nijmegen, the Netherlands; b Radboud University Medical Center, Department of Medical Psychology, Nijmegen, the Netherlands; c Radboud University Medical Center, Department of Pulmonary Diseases, Nijmegen, the Netherlands; d Radboud University Medical Center, Radboud Institute for Molecular Life Sciences, Nijmegen, the Netherlands; e Radboud University, Donders Institute for Brain, Cognition and Behaviour, Nijmegen, the Netherlands; f Vincent van Gogh Institute for Psychiatry, Venray, the Netherlands

**Keywords:** COVID-19, Respiratory distress syndrome, Cognitive dysfunction, Neuroinflammation, Immunomodulatory therapy, Long COVID

## Abstract

**Background and objectives:**

Long-term cognitive performance data in former critically ill COVID-19 patients are sparse. Current evidence suggests that cognitive decline is related to neuroinflammation, which might be attenuated by COVID-19 related anti-inflammatory therapies. The objective of this prospective cohort study was to study long term cognitive outcomes following severe COVID-19 and the relation to anti-inflammatory therapies.

**Methods:**

Prospective observational cohort of patients that survived an intensive care unit (ICU) admission due to severe COVID-19. Six months after hospital discharge, we extensively assessed both objective cognitive functioning and subjective cognitive complaints. Furthermore, patients were stratified in cohorts according to their anti-inflammatory treatment (i.e. no immunomodulatory therapy, dexamethasone, or both dexamethasone and interleukin-6 receptor antagonist tocilizumab).

**Results:**

96 patients were included (March 2020–June 2021, median [IQR] age 61 [55–69] years). 91% received invasive mechanical ventilation, and mean ± SD severity-of-disease APACHE–II–score at admission was 15.8 ± 4.1. After 6.5 ± 1.3 months, 27% of patients scored cognitively impaired. Patients that did or did not develop cognitive impairments were similar in ICU-admission parameters, clinical course and delirium incidence. Patients with subjective cognitive complaints (20%) were more likely women (61% vs 26%), and had a shorter ICU stay (median [IQR] 8 [5–15] vs 18 [9–31], p = 0.002). Objective cognitive dysfunction did not correlate with subjective cognitive dysfunction. 27% of the participants received dexamethasone during intensive care admission, 44% received additional tocilizumab and 29% received neither. Overall occurrence and severity of cognitive dysfunction were not affected by anti-inflammatory therapy, although patients treated with both dexamethasone and tocilizumab had worse executive functioning scores (Trail Making Test interference) than patients without anti-inflammatory treatment (T-score 40.3 ± 13.5 vs 49.1 ± 9.3, p = 0.007).

**Discussion:**

A relevant proportion of critically ill COVID-19 patients shows deficits in long-term cognitive functioning. Apart from more pronounced executive dysfunction, overall, anti-inflammatory therapy appeared not to affect long-term cognitive performance. Our findings provide insight in long-term cognitive outcomes in patients who survived COVID-19, that may facilitate health-care providers counseling patients and their caregivers.

## Highlights

• Up to a third of survivors of severe COVID-19 develops long-term cognitive impairment.
• Subjective cognitive complaints six months following severe COVID-19 do not correlate with objective cognitive impairment.
• COVID-19 related immunomodulatory therapy is not associated with the overall incidence of cognitive dysfunction.

## 1 Introduction

Impaired cognitive functioning and mental health deficits are common in survivors of critical illness (Wilcox et al., 2013; Iwashyna et al., 2010). Now that we are faced with a large number of patients that survived intensive care unit (ICU) admissions related to coronavirus disease 2019 (COVID-19) (Huang et al., 2021), data on long-term neuropsychological perspectives are needed to facilitate counseling of patients and their families and to setup sufficient provisions for cognitive neurorehabilitation. Available studies on cognitive outcomes in patients with COVID-19 have focused on short-term cognitive impairment after mild, or moderate COVID-19 (Pistarini et al., 2021; Alemanno et al., 2021; Beaud et al., 2021), which has a higher risk of being influenced by residual physical impairments. Less is known about the long-term and domain-specific neuropsychological outcomes in survivors of COVID-19 related critical illness.

Systemic inflammation can lead to disruption of the blood-brain barrier causing increased cytokine levels within the brain and microglial activation inducing a neuroinflammatory response (van Gool et al., 2010; Semmler et al., 2005, 2008; Westhoff et al., 2019; Lopez-Rodriguez et al., 2021). Neuroinflammation is assumed to be related to the subsequent development of cognitive impairments - as demonstrated in both pre-clinical (Wan et al., 2007; Semmler et al., 2007; Cao et al., 2010) and clinical research (Barichello et al., 2019; Shen et al., 2019; Bradburn et al., 2019; Singh-Manoux et al., 2014) - and every sepsis episode increases the risk of developing dementia (Peters van Ton et al., 2022). In severe COVID-19, a prolonged systemic inflammatory state might cause similar effects, along with the interaction of SARS-CoV-2 with ACE2 receptors on neurons, potentially causing additional axonal damage (Amruta et al., 2021). Both post-mortem (Matschke et al., 2020) and *in vivo* (Lopez-Rodriguez et al., 2021) analyses in patients with COVID-19 showed diffuse neuroinflammation.

During the course of the pandemic, anti-inflammatory drugs were introduced that improved the survival of patients with severe COVID-19 acute respiratory distress syndrome (ARDS). Initially, dexamethasone (Recovery Collaborative Group Horby et al., 2021), and subsequently interleukin-6 (IL-6) receptor antagonists (i.e. tocilizumab) were added (Investigators et al., 2021). Hypothetically, these anti-inflammatory drugs might attenuate a neuroinflammatory state. Corticosteroids such as dexamethasone have strong anti-inflammatory effects within the brain, and are widely used in patients with cerebral vasculitis or in patients with cerebral edema related to brain tumors (Dietrich et al., 2011). Research on tocilizumab and its effect on neuropsychiatric symptoms has shown inconsistent results, varying from positive effects (Figueiredo-Braga et al., 2018), absent effects (Girgis et al., 2018) and even negative effects (Lee et al., 2014; Knight et al., 2021). Therefore, the possible impact of dexamethasone and/or IL-6 receptor antagonists on long-term neuropsychological outcomes in COVID-19 patients remains unknown.

The first objective of this study was to assess long-term cognitive function and mental wellbeing in ICU survivors who suffered from severe COVID-19. The second objective was to study whether anti-inflammatory treatment with dexamethasone and IL-6 receptor antagonists were associated with long-term cognitive outcomes.

## 2 Methods

### 2.1 Study design and population

**Fig. 1:**
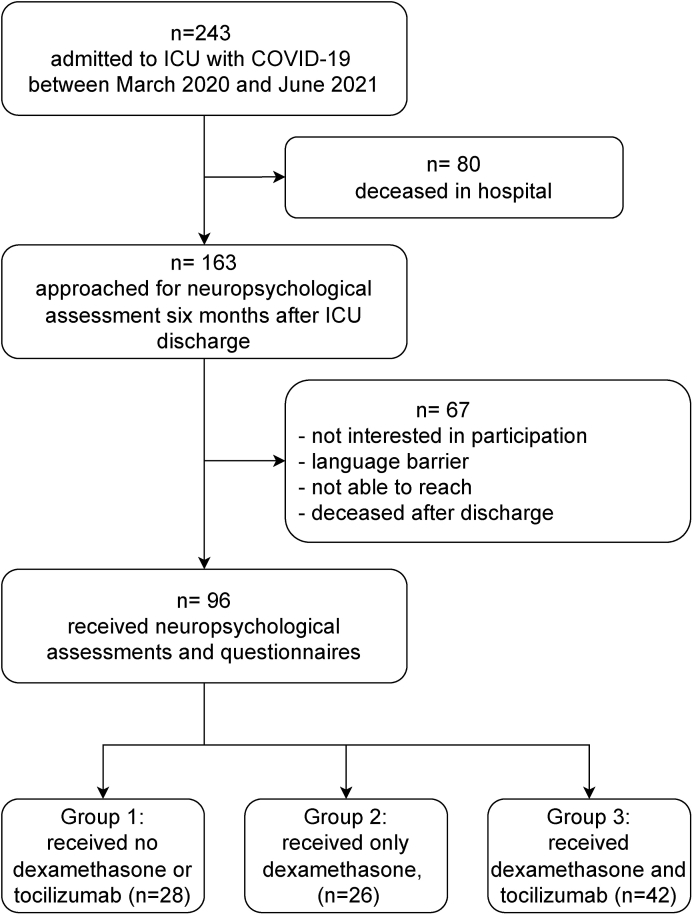
Flowchart of inclusion procedure. **Abbreviations:** ICU, intensive care unit; COVID-19, coronavirus disease 2019.

This is a prospective single center longitudinal cohort study, performed at the Radboud University Medical Center, Nijmegen, The Netherlands. We screened all consecutive adult patients admitted to the ICU with COVID-19 (confirmed through real-time PCR analysis of nasal and throat swab specimens), between March 18, 2020 and June 6, 2021. In case of survival, patients that were fluent in Dutch were approached after six months to participate (Fig. 1).

We stratified the participants into three groups based on whether they received COVID-19 related anti-inflammatory medication. The first group received neither dexamethasone nor IL-6 receptor antagonist tocilizumab (RoActemra, XGVS Roche, The Netherlands), and was admitted between March 18, 2020 and April 27, 2020. The second group received dexamethasone (once daily 6 mg intravenous for ten consecutive days), and was admitted between October 6, 2020 and March 30, 2021. The third group received both dexamethasone (once daily 6 mg intravenous for ten consecutive days) and tocilizumab (single intravenous dose 400–800 mg, 8 mg/kg) and was admitted between November 23, 2020 and June 6, 2021.

### 2.2 Clinical measures

Baseline characteristics (sex, age, body mass index (BMI), medical history), COVID-19 specific data (date of onset of symptoms, disease severity scores (APACHE II (Knaus et al., 1985) and SOFA (Vincent et al., 1996)) and laboratory values) were prospectively collected in a research database. Clinical course and relevant outcomes were additionally collected from medical records. Acute respiratory distress syndrome (ARDS) was diagnosed and classified into mild, moderate and severe following the Berlin definition for ARDS (Ards Definition Task Force Ranieri et al., 2012).

Coma was assessed using the Richmond Agitation Sedation Scale (RASS) (Sessler et al., 2002) and defined as RASS ≤ -3. Delirium was assessed using the Confusion Assessment Method for the Intensive Care Unit (CAM-ICU) (Ely et al., 2001). Delirium- and coma-free days were defined as the number of ICU days during which the patient was without delirium and not in coma. Laboratory values at day of ICU admission were collected. In case of missing laboratory values at the day of admission, we used the first available measurement (up to a maximum of two days after admission).

### 2.3 Extensive neuropsychological test battery and questionnaires

Four trained psychologists performed neuropsychological evaluations. The Montreal Cognitive Assessment (MoCA) was used to detect cognitive abnormalities (Nasreddine et al., 2005). The Trail Making Test (TMT; parts A and B, and the interference score B/A correcting for baseline speed) (Partington and Leiter, 1949; Reitan, 1958; Bowie and Harvey, 2006), Letter Digit Substitution Test (Natu and Agarwal, 1995), and the Digit Span (Kessels et al., 2011; Wechsler, 2012) were administered to detect subtle abnormalities in the cognitive domains executive functioning and information processing speed. To estimate premorbid intelligence level, the Dutch version of the National Adult Reading Test (NART-IQ) was used (Nelson and O'Connell, 1978). This test was added to the study protocol later during the study and is therefore missing for patients who participated prior to April 1, 2021. Level of education (7-point rating scale based on the Dutch educational system) was divided into three ordinal categories: low educational level (levels 1–4), average educational level (level 5), and high educational level (levels 6–7) (Verhage, 1964).

We compared individual neuropsychological test results to available Dutch normative data, resulting in standardized age-, sex-, and education-adjusted T-scores (M = 50, SD = 10). Also, we converted the MoCA results to T-scores corrected for sex, age and education, based on data from healthy Dutch controls (de Vent et al., 2016; Kessels et al., 2022). Consequently, an *overall T-score* was calculated by averaging the T-scores of all cognitive tests. T-scores of each individual test were classified as following. ‘*Normal’* performance was defined as performance above −1 SD from the normative mean. ‘*Below average’* as between −1 SD and −1.5 SD from the normative mean and ‘*Impaired*’ as below −1.5 SD from the normative mean (the lowest 6.7% of the normal population) (Jak et al., 2009). Ultimately, patients were dichotomized into two groups: cognitively unimpaired or cognitively impaired. Patients would classify as cognitively impaired, when they categorized as *impaired* on two or more of the cognitive tests.

Apart from objective neuropsychological assessments, we asked participants to fill out self-report questionnaires about their subjective physical and mental wellbeing. In order to assess subjective cognitive state (i.e. experiencing lapses of concentration, memory, and/or cognition), patients were divided into two groups based on their Cognitive Failure Questionnaire (CFQ) score (Broadbent et al., 1982). Patients with high (total score of 44–54) and very high scores (total score of >54) were classified as having cognitive complaints. Additionally, we assessed psychological distress symptoms using the Hospital Anxiety and Depression Scale (HADS) (Zigmond and Snaith, 1983). For both HADS Anxiety and HADS Depression, a cut off score of ≥8 was considered indicative of severe symptoms of anxiety or depression. Frailty was scored using the Clinical Frailty Scale (CFS, version 2.0) (Church et al., 2020), resulting in a summarized score of 1–9, with a higher score reflecting higher frailty. General health status was assessed with the SF-12 (version 1.0) (Ware et al., 1996). Finally, patients were asked about psychopathological symptoms using the Brief Symptom Inventory (BSI) (Lani and Derogatis, 2010).

### 2.4 Statistical analysis

Statistics were performed using IBM-SPSS software (version 25) and GraphPad Prism 9. Alpha was set at 0.05 for all analyses. Continuous variables were presented as mean ± standard deviation (SD) or median [first and third inter quartile range, IQR]. Categorical variables were presented as frequency (n) with proportions (%). Normality of data distribution was checked using Normal Q-Q-plots, and homogeneity using Levene's test. Comparisons between continuous variables in groups were made by independent-samples T-test, Mann-Whitney-U test, one-way analysis of variance (ANOVA), or Kruskal-Wallis test, as relevant. If statistically significant between-group differences occurred, Bonferroni corrected post-hoc tests were performed. Categorical comparisons between groups were analyzed using chi-square test. We used Pearson's correlation to examine the relationship between subjective and objective cognition.

### 2.5 Standard protocol approvals, registrations and patient consents

The Dutch Medical Research Ethics Committee, region Arnhem-Nijmegen (CMO, 2020–6816) approved this study, which was conducted according to the principles of the Declaration of Helsinki. All participants provided written informed consent.

### 2.6 Data availability

The anonymized datasets generated and/or analyzed during the current study are available from the corresponding author on reasonable request.

## 3 Results

### 3.1 Baseline characteristics

A total of 96 consecutive patients with severe COVID-19 were included in the study (Fig. 1). The median age was 61 (IQR 55–69) years and 64 (67%) patients were men. All patients met the Berlin Criteria for ARDS (Ards Definition Task Force Ranieri et al., 2012), and 87 (91%) received invasive mechanical ventilation. All underwent a full neuropsychological assessment and 92 filled out the self-reporting questionnaires. Cognitive tests were performed at a mean ± SD of 6.5 ± 1.3 months after ICU discharge. Patient characteristics are shown in Table 1.

**Table 1:** Demographics, baseline characteristics and outcomes of all patients.

	Full study sample (n = 96)	No anti-inflammatory treatment (n = 28)	Dexamethasone (n = 26)	Dexamethasone and tocilizumab (n = 42)	p-value
Demographics					
Age, median [IQR], years	61 [55–69]	62 [58–70]	60 [51–67]	63 [54–69]	p = 0.33
Male sex, n (%)	64 (66.7)	19 (67.9)	18 (69.2)	27 (64.3)	p = 0.90
BMI, median [IQR], kg/m^2^	28.3 [25.9–31.5]	27.1 [24.4–29.2]^†‡^	29.3 [27.0–32.4]*	29.4 [26.2–32.2]*	**p = 0.015**
Level of education, n (%)					
- Low	37 (38.5)	11 (39.3)	10 (38.5)	16 (38.1)	p = 0.57
- Average	31 (32.3)	6 (21.4)	9 (34.6)	16 (38.1)	
- High	28 (29.2)	11 (39.3)	7 (26.9)	10 (23.8)	
NART-IQ, mean (SD)	94.4 (16.3)	*not available*	93.7 (15.1)	94.8 (17.2)	p = 0.79
Time from first COVID-19 symptoms to ICU admission, mean (SD), days	11.3 (4.5)	11.4 (4.6)	11.1 (6.3)	11.4 (3.1)	p = 0.98
Time from ICU discharge to cognitive test, mean (SD), months	6.5 (1.3)	6.7 (0.8)^†‡^	7.7 (1.2)*^‡^	5.7 (1.1)*^†^	**p < 0.001**
**Medical history, n (%)**					
No comorbidities	9 (9.4)	9 (32.1)^†‡^	0 (0.0)*^‡^	0 (0.0)*^†^	**p < 0.001**
Hypertension	45 (46.9)	15 (53.6)	10 (38.5)	20 (47.6)	p = 0.54
Diabetes Mellitus	22 (22.9)	5 (17.9)	6 (23.1)	11 (26.2)	p = 0.72
COPD	10 (10.4)	2 (7.1)	5 (19.2)	3 (7.1)	p = 0.23
Renal dialysis	0 (0.0)	0 (0.0)	0 (0.0)	0 (0.0)	NA
Immunological insufficiency	5 (5.2)	4 (14.3)^‡^	0 (0.0)	1 (2.4)*	**p = 0.034**
**At admission to ICU**					
APACHE II, mean (SD)	15.8 (4.1)	15.2 (5.2)	15.7 (3.9)	16.2 (3.5)	p = 0.60
SOFA, mean (SD)	5.8 (2.6)	6.2 (2.6)	5.6 (3.0)	5.6 (2.4)	p = 0.64
Temperature, median [IQR], °C	37.3 [36.7–38.2]	38.3 [37.5–38.9]^†‡^	37.3 [36.7–38.0]*	37.0[36.5–37.6]*	**p < 0.001**
Invasive Mechanical ventilation, n (%)	87 (90.6)	28 (100.0)^‡^	26 (100.0)^‡^	33 (78.6)*^†^	**p = 0.002**
FiO2, median [IQR], % oxygen	63 [53–80]	60 [55–80]	70 [55–90]	65 [50–76]	p = 0.42
PaO2/FiO2 ratio, median [IQR]	135 [101–180]	144 [120–189]	147 [84–206]	127 [98–162]	p = 0.30
ARDS classification, n (%)					
- Mild	18 (18.8)	6 (21.4)	6 (23.1)	6 (14.3)	p = 0.21
- Moderate	54 (56.3)	19 (67.9)	11 (42.3)	25 (57.1)	
- Severe	24 (25)	3 (10.7)	9 (34.6)	12 (28.6)	
**Laboratory results at admission**					
CRP, median [IQR], mg/L	95 [47–171]	190 [127–269]^†‡^	75 [44–99]*	65 [27–136]*	**p < 0.001**
PCT, median [IQR], μg/L	0.29 [1.12–0.50]	0.52 [0.31–0.95]^†‡^	0.28 [0.09–0.38]*	0.21[0.10–0.36]*	**p < 0.001**
Ferritin, median [IQR], μg/L	1345 [701-1905]	1446 [709-2182]	1355 [741-2327]	1285 [645-1904]	p = 0.85
D-dimer, median [IQR], μg/L	1835 [917-4245]	2980 [1475–15,313]^†^	1340 [635-2012]*	1735 [980-4025]	**p = 0.004**
Creatinin, median [IQR], μmol/L	75 [56–93]	79 [63–100]	74 [60–87]	75 [55–89]	p = 0.52
Leukocytes, median [IQR], 10^9^/L	9.4 [6.7–13.0]	8.2 [6.2–12.8]^†^	12.6 [9.5–15.4]*^‡^	8,2 [6.5–11.1]^†^	**p = 0.002**
**Outcomes**					
Time on ventilator, median [IQR], days	13 [6–23]	20 [12–27]^‡^	8 [5–27]	12 [4–19]*	**p = 0.011**
Duration of ICU stay, median [IQR], days	16 [9–28]	23 [12–30]	10 [7–28]	15 [8–26]	p = 0.076
Coma during ICU stay, n (%)	87 (90.6)	28 (100.0)^‡^	25 (96.1)^‡^	34 (80.9)*^†^	**p = 0.015**
Delirium during ICU stay, n (%)	70 (72.9)	22 (78.6)	19 (73.1)	29 (69.0)	p = 0.68
Delirium or coma, median [IQR], days	11 [5–20]	14 [10–19]	8 [4–23]	9 [4–21]	p = 0.18
Delirium- and coma-free, median [IQR], days	5 [3–9]	6 [4–11]	3 [2–6]	5 [3–8]	p = 0.098
Secondary infections, n (%)	21 (21.9)	7 (25.0)	5 (19.2)	9 (21.4)	p = 0.87
**Immunomodulating therapy during hospital stay**					
Dexamethasone, n (%)	68 (70.8)	0 (0.0)^†‡^	26 (100.0)*	42 (100.0)*	**p < 0.001**
Tocilizumab, n (%)	42 (43.8)	0 (0.0)^‡^	0 (0.0)^‡^	42 (100.0)*^†^	**p < 0.001**
(Hydroxy)chloroquine, n (%)	28 (29.2)	28 (100.0)^†‡^	0 (0.0)*	0 (0.0)*	**p < 0.001**

Significant p-values of between-group differences, were followed by Bonferroni-corrected post-hoc analyses.

*p ≤ 0.05 vs no anti-inflammatory treatment group; †p ≤ 0.05 vs dexamethasone group; ‡p ≤ 0.05 vs tocilizumab and dexamethasone group.

**Abbreviations:** COVID-19, coronavirus disease 2019; ICU, intensive care unit; SD, standard deviation; IQR, interquartile range; NA, not applicable; BMI, body mass index; NART, National Adult Reading Test; IQ, intelligence quotient; COPD, Chronic Obstructive Pulmonary Disease; APACHE II, Acute Physiology and Chronic Health Evaluation II; SOFA, Sequential Organ Failure Assessment; PaO2, partial pressure of oxygen; FiO2, fraction of inspired oxygen; ARDS, Acute Respiratory Distress Syndrome; CRP, C-reactive protein; PCT, procalcitonin.

**ARDS classification:** mild: PaO2/FiO2 ratio 200–300; moderate: PaO2/FiO2 ratio 100–200; severe: PaO2/FiO2 ratio <100

### 3.2 Objective cognitive outcome

**Fig. 2:**
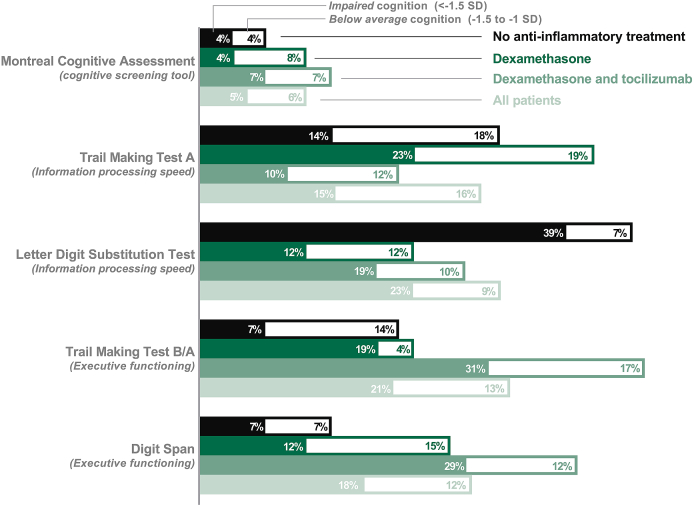
Percentages of impaired or below average outcomes on cognitive tests in patients with severe COVID-19 six months after discharge from the intensive care unit, stratified by anti-inflammatory treatment.

Overall, 26 participants (27%) were classified as cognitively impaired based on their test results. More specifically, the (age-, sex- and education corrected (Kessels et al., 2022)) MoCA was impaired (T-score < -1.5 SD) in 5% of patients. On executive functioning tests, 21% of patients scored impaired on TMT-B/A, and 18% on the Digit Span test. Information processing performances (LDST and TMT-A) were impaired in respectively 23% and 15% of patients. Fig. 2 shows the proportions of unimpaired, below average, and impaired cognition for each cognitive test. Further details of all neuropsychological test results with the corresponding standardized T-scores and results of the self-report questionnaires can be found in Table 2.

**Table 2:** Results of neuropsychological tests and self-report questionnaires.

	Full study sample (n = 96)	No anti-inflammatory treatment (n = 28)	Dexamethasone (n = 26)	Dexamethasone and tocilizumab (n = 42)	p-value
**Neuropsychological tests**					
MoCA raw score, median [IQR]	26 [24–27]	26 [25–28]	25 [24–27]	26 [24–27]	p = 0.44
MoCA T-score, mean (SD)	50.5 (9.3)	52.8 (9.4)	48.7 (9.3)	50.1 (9.3)	p = 0.26
TMT-A T- score, mean (SD)	48.1 (14.1)	45.1 (13.0)	44.7 (14.6)	52.2 (13.8)	**p = 0.042**^§^
TMT-B T-score, mean (SD)	46.3 (11.2)	47.0 (10.1)	46.3 (11.5)	42.6 (14.5)	p = 0.35
TMT-B/A T- score, mean (SD)	46.3 (11.6)	49.1 (9.3)^‡^	45.1 (12.8)	40.3 (13.5)*	**p = 0.007**
Digit Span T- score, mean (SD)	46.5 (10.9)	49.5 (9.2)	45.4 (8.9)	45.2 (12.7)	p = 0.23
LDST T-score, mean (SD)	45.0 (11.2)	41.3 (12.0)	45.0 (9.2)	47.5 (11.4)	p = 0.075
Overall T-score, mean (SD)	47.0 (7.5)	47.6 (6.2)	46.2 (6.8)	47.1 (8.7)	p = 0.81
**Weighted test results**					
MoCA, n (%)					
- Unimpaired	85 (88.5)	26 (92.9)	23 (88.5)	36 (85.7)	p = 0.90
- Below average	6 (6.3)	1 (3.6)	2 (7.7)	3 (7.1)	
- Impaired	5 (5.2)	1 (3.6)	1 (3.8)	3 (7.1)	
TMT-A, n (%)					
- Unimpaired	67 (69.8)	19 (67.9)	15 (57.7)	33 (78.6)	p = 0.44
- Below average	15 (15.6)	5 (17.9)	5 (19.2)	5 (11.9)	
- Impaired	14 (14.6)	4 (14.3)	6 (23.1)	4 (9.5)	
TMT-B, n (%)					
- Unimpaired	66 (68.8)	21 (75.0)	19 (73.1)	26 (61.9)	p = 0.55
- Below average	8 (8.3)	3 (10.7)	2 (7.7)	3 (7.1)	
- Impaired	22 (22.9)	4 (14.3)	5 (19.2)	13 (31.0)	
TMT-B/A, n (%)					
- Unimpaired	64 (66.7)	22 (78.6)	20 (76.9)	22 (52.4)	p = 0.058
- Below average	12 (12.5)	4 (14.3)	1 (3.8)	7 (16.7)	
- Impaired	20 (20.8)	2 (7.1)	5 (19.2)	13 (31.0)	
Digit Span, n (%)					
- Unimpaired	68 (70.8)	24 (85.7)	19 (73.1)	25 (59.5)	p = 0.11
- Below average	11 (11.5)	2 (7.1)	4 (15.4)	5 (11.9)	
- Impaired	17 (17.7)	2 (7.1)	3 (11.5)	12 (28.6)	
LDST, n (%)					
- Unimpaired	65 (67.7)	15 (53.6)	20 (76.9)	30 (71.4)	p = 0.16
- Below average	9 (9.4)	2 (7.1)	3 (11.5)	4 (9.5)	
- Impaired	22 (22.9)	11 (39.3)	3 (11.5)	8 (19.0)	
**Dichotomized cognitive outcome, n (%)**					
- Unimpaired	70 (72.9)	23 (82.1)	19 (73.1)	28 (66.7)	p = 0.36
- Cognitively impaired	26 (27.1)	5 (17.9)	7 (26.9)	14 (33.3)	
**Self-reporting Questionnaires**					
CFQ total, mean (SD)	33.0 (15.2)	32.4 (14.1)	36.2 (15.2)	31.3 (16.2)	p = 0.44
CFS, median [IQR]	3 [2–4]	3 [2–5]	3 [2–4]	3 [2–4]	p = 0.82
HADS, median [IQR]					
- Total score	8 [4–15]	8 [4–16]	8 [6–15]	8 [4–15]	p = 0.91
- Anxiety score	4 [2–8]	4 [2–8]	4 [3–8]	3 [2–7]	p = 0.56
- Depressive score	4 [2–7]	3 [2–7]	3 [2–7]	4 [2–7]	p = 0.87
SF-12, median [IQR]					
- Physical T-score	38.0 [31.0–46.8]	38.0 [34.2–42.1]	37.0 [30.2–47.0]	39.8 [26.7–49.4]	p = 0.65
- Mental T-score	52.0 [41.3–57.8]	53.5 [41.1–59.4]	51.7 [41.1–57.0]	51.6 [42.0–57.8]	p = 0.46
BSI (GSI) T-score, median [IQR]	49.7 [37.6–59.9]	48.7 [35.2–59.9]	54.1 [44.0–61.7]	47.1 [37.6–59.6]	p = 0.43

All individual cognitive test results were corrected for age, sex and education, using normative data of a healthy Dutch control population. Significant p-values of between-group differences, were followed by Bonferroni-corrected post-hoc analyses. *p ≤ 0.05 vs no anti-inflammatory treatment group; †p ≤ 0.05 vs dexamethasone group; ‡p ≤ 0.05 vs tocilizumab and dexamethasone group; § post hoc analysis showed no statistically significant differences between any of the groups.

**Weighted test results**: *unimpaired*: T-score >40 (>−1 SD); *below average*: T-score 35–40 (−1.5 to −1 SD); *impaired*: T-score <40 (<−1.5 SD). **Dichotomization**: a patient scored “cognitively impaired” when he/she had a weighted score of *impaired* on two or more tests. **Abbreviations:** MoCA, Montreal Cognitive Assessment; TMT, Trail Making Test; Digit Span, Wechsler Adult Intelligence Scale-IV Digit Span test; LDST, Letter Digit Substitution Test. CFQ, Cognitive Failure Questionnaire; CFS, Clinical Frailty Scale; HADS, Hospital Anxiety and Depression Scale; SF-12, Short Form Health Survey-12; BSI, Brief Symptom Inventory; GSI, Global Severity Index.

When comparing cognitively impaired with unimpaired patients, no significant differences were observed in age, sex, BMI, time from first COVID-19 symptoms to ICU admission, and time from ICU discharge until cognitive testing (Table 3). Apart from the finding that patients with cognitive impairment were 4.3 times more likely to have diabetes mellitus (p < 0.001), laboratory results at ICU admission, ICU length of stay, and delirium- and/or coma-free days did not differ significantly between the groups. Furthermore, the cognitively impaired patients showed a similar clinical frailty score after six months and reported similar anxiety and depressive symptoms, general health status, and overall psychopathological symptoms compared to patients that were not cognitively impaired (Table s1 of supplemental material).

**Table 3:** Baseline characteristics and outcomes in patients with and without objective cognitive deficits or subjective cognitive complaints.

	Objective cognition			Subjective cognition		
	Unimpaired (n = 70)	Impaired (n = 26)	p-value	Unimpaired (n = 74)	Impaired (n = 18)	p-value
**Demographics**						
Age, median [IQR], years	61 [54–69]	64 [56–69]	p = 0.32	64 [57–70]	60 [53–63]	p = 0.070
Male sex, n (%)	46 (65.7)	18 (69.2)	p = 0.75	55 (74.3)	7 (38.9)	**p = 0.002**
BMI, median [IQR], kg/m^2^	28.7 [25.8–31.1]	27.5 [26.1–33.5]	p = 0.65	28.4 [26.0–30.9]	28.4 [24.3–38.9]	p = 0.63
Level of education, n (%)						
- Low	21 (30.0)	16 (61.5)	p = 0.011*	32 (43.2)	3 (16.7)	**p = 0.033**^†^
- Average	24 (34.3)	7 (26.9)		19 (25.7)	10 (55.6)	
- High	25 (35.7)	3 (11.5)		23 (31.1)	5 (27.8)	
NART-IQ, mean (SD)	97.4 (15.3)	87.2 (16.9)	**p = 0.021**	95.3 (16.5)	95.1 (13.1)	p = 0.96
Time between first COVID-19 symptoms to ICU admission, mean (SD), days	11.4 (4.6)	11.0 (4.2)	p = 0.93	11.5 (4.6)	10.2 (4.0)	p = 0.26
Time from ICU discharge to cognitive test, mean (SD), months	6.7 (1.3)	6.1 (1.4)	p = 0.062	6.5 (1.2)	6.9 (1.5)	p = 0.18
**Medical history, n (%)**						
No comorbidities	8 (11.4)	1 (3.8)	p = 0.26	8 (10.8)	0 (0.0)	p = 0.14
Hypertension	29 (41.4)	16 (61.5)	p = 0.079	39 (52.7)	5 (27.8)	p = 0.058
Diabetes Mellitus	9 (12.9)	13 (50.0)	**p < 0.001**	18 (24.3)	3 (16.7)	p = 0.49
COPD	7 (10.0)	3 (11.5)	p = 0.83	7 (9.5)	3 (16.7)	p = 0.38
Renal dialysis	0 (0.0)	0 (0.0)	NA	0 (0.0)	0 (0.0)	NA
Immunological insufficiency	11 (15.7)	4 (15.4)	p = 0.97	11 (14.9)	3 (16.7)	p = 0.85
**At admission to ICU**						
APACHE II, mean (SD)	15.7 (4.4)	16.0 (3.4)	p = 0.76	16.1 (4.4)	14.4 (2.7)	p = 0.15
SOFA, mean (SD)	5.8 (2.7)	5.6 (2.6)	p = 0.71	5.9 (2.4)	5.6 (3.4)	p = 0.69
Temperature, median [IQR], °C	37.4 [36.7–38.5]	37.2 [36.8–37.7]	p = 0.41	37.2 [36.7–38.5]	37.3 [36.8–38.2]	p = 0.69
FiO2, median [IQR], %	60 [55–80]	68 [51–76]	p = 0.99	63 [55–80]	58 [49–71]	p = 0.20
PaO2/FiO2 ratio, median [IQR]	133 [101–186]	144 [101–178]	p = 0.70	134 [102–170]	169 [94–225]	p = 0.25
ARDS classification, n (%)						
- Mild	13 (18.6)	5 (19.2)	p = 0.95	10 (13.5)	7 (38.9)	**p = 0.027**^‡^
- Moderate	40 (57.1)	14 (53.8)		46 (62.2)	6 (33.3)	
- Severe	17 (24.3)	7 (26.9)		18 (24.3)	5 (27.8)	
**Laboratory results at admission**						
CRP, median [IQR], mg/L	98 [53–209]	73 [29–136]	p = 0.054	97 [50–175]	69 [40–187]	p = 0.53
PCT, median [IQR], μg/L	0.28 [0.11–0.49]	0.32 [0.18–0.54]	p = 0.48	0.31 [0.13–0.58]	0.18 [0.10–0.30]	**p = 0.017**
Ferritin, median [IQR], μg/L	1345 [703-2536]	1335 [589-1797]	p = 0.40	1473 [954–2211]	885 [580–1227]	**p = 0.012**
D-dimer, median [IQR], μg/L	1790 [827-4405]	2575 [932-4470]	p = 0.58	1970 [913–5520]	1475 [843–2240]	p = 0.13
Creatinin, median [IQR], μmol/L	75 [56–89]	78 [62–128]	p = 0.31	76 [63–95]	60 [50–81]	**p = 0.018**
Leukocytes, median [IQR], 10^9^/L	9.5 [6.4–12.9]	8.8 [7.2–13.3]	p = 0.92	9.5 [6.8–13.6]	9.5 [6.1–11.3]	p = 0.34
**Outcomes**						
Mechanical ventilation, n (%)	62 (88.6)	25 (96.2)	p = 0.26	67 (90.5)	16 (88.9)	p = 0.83
Time on ventilator, median [IQR], days	13 [6–23]	16 [7–27]	p = 0.42	15 [7–27]	8 [2–14]	**p = 0.015**
Duration of ICU stay, median [IQR], days	15 [8–28]	18 [10–30]	p = 0.29	18 [9–31]	8 [5–15]	**p = 0.002**
Coma during ICU stay, n (%)	62 (88.6)	25 (96.2)	p = 0.26	68 (91.9)	15 (83.3)	p = 0.27
Delirium during ICU stay, n (%)	48 (68.6)	22 (84.6)	p = 0.12	55 (74.3)	11 (61.1)	p = 0.26
Delirium or coma, median [IQR], days	11 [4–19]	14 [6–24]	p = 0.23	13 [5–22]	6 [2–13]	**p = 0.016**
Delirium- and coma-free, median [IQR], days	4 [3–9]	5 [3–7]	p = 0.81	5 [3–10]	3 [1–4]	**p = 0.002**
Secondary infections, n (%)	14 (20.0)	7 (26.9)	p = 0.47	18 (24.3)	2 (11.1)	p = 0.22
**Immunomodulating therapy during hospital stay**						
Dexamethasone, n (%)	47 (67.1)	21 (80.8)	p = 0.19	52 (70.3)	14 (73.7)	p = 0.77
Tocilizumab, n (%)	28 (40.0)	14 (53.8)	p = 0.22	33 (44.6)	7 (36.8)	p = 0.54
(Hydroxy)chloroquine, n (%)	23 (32.9)	5 (19.2)	p = 0.19	22 (29.7)	5 (27.8)	p = 0.87

*post hoc analysis with Bonferroni correction revealed a significant difference in % low-educated participants (p = 0.005). † After Bonferroni correction, statistical significant differences were lost. ‡ post hoc analysis with Bonferroni correction revealed a significant difference in % mild ARDS (p = 0.013).

**Abbreviations:** COVID-19, coronavirus disease 2019; ICU, intensive care unit; SD, standard deviation; IQR, interquartile range; NA, not applicable; BMI, body mass index; NART, National Adult Reading Test; IQ, intelligence quotient; COPD, chronic obstructive pulmonary disease; APACHE II, Acute Physiology and Chronic Health Evaluation II; SOFA, Sequential Organ Failure Assessment; PaO2, partial pressure of oxygen; FiO2, fraction of inspired oxygen; ARDS, Acute Respiratory Distress Syndrome; CRP, C-reactive protein; PCT, procalcitonin. **ARDS classification:** mild: PaO2/FiO2 ratio 200–300; moderate: PaO2/FiO2 ratio 100–200; severe: PaO2/FiO2 ratio <100.

### 3.3 Subjective cognitive outcome

Subjective cognitive health was assessed through the Cognitive Failure Questionnaire (CFQ), resulting in 18 (20%) patients classifying as having severe cognitive complaints. Clinical characteristics between patients with and without cognitive complaints are specified in Table 3.

The patients reporting subjective cognitive deficits had a seven-day shorter time on ventilator (median [IQR] 8 [2–14] vs 15 [7–27], p = 0.015) and a ten day shorter ICU length of stay (median 8 [5–15] vs 18 [9–31], p = 0.002). Delirium incidence in patients with cognitive complaints was 61% and did not significantly differ from patients without complaints (74%, p = 0.264). Regarding the other questionnaires, the subjectively cognitively affected group reported 2.7 times higher anxiety scores (p < 0.001) and more psychopathological symptoms (median BSI T-score 63.7 [58.2–68.9] vs 47.1 [36.4–56.8], p < 0.001). Likewise, their self-reported health status was lower compared to patients without cognitive complaints, both in the SF-12 physical (median T-score 32.9 [23.0–39.4] vs 39.3 [32.1–48.1], p = 0.026) as the mental domain (median T-score 43.0 [37.8–51.3] vs 53.8 [46.5–59.1], p = 0.004). Further details about test-results can be found in Supplementary Table S1. We did not find a significant correlation between subjective cognitive function (CFQ score) and the mean overall T-score of cognitive tests or any of the individual objective cognitive test results. Of the patients classified as having objective cognitive impairment, 3 (13%) reported subjective cognitive complaints, while 15 (22%) of the objectively unimpaired group reported cognitive complaints (p = 0.310).

### 3.4 Anti-inflammatory therapy

The study population was divided into patients who received no immune-modulating therapy (n = 28, 29%), patients who received only dexamethasone (n = 26, 27%), and patients who received both dexamethasone and IL-6 receptor antagonist tocilizumab (n = 42, 44%). Baseline characteristics and clinical outcomes for each group are shown in Table 1. There were no differences in age, sex, level of education, disease severity at admission, or ARDS severity between the groups. C-reactive protein at ICU admission was significantly higher in patients that did not receive anti-inflammatory therapy (median [IQR] 190 [127–269], compared to the other groups (75 [44–99] and 65 [27–136], respectively, p < 0.001). Similar patterns were observed for procalcitonin (p < 0.001) and D-dimer (p = 0.004). All patients who did not receive immunomodulatory therapy, as well as those in the dexamethasone group, received invasive mechanical ventilation, compared to 78% of the patients that received dexamethasone and tocilizumab (p = 0.002). There were no differences in delirium- and coma-free days between the treatment groups.

### 3.5 Effect of anti-inflammatory therapy on cognitive function

Fig. 2 also shows the cognitive test results between the different anti-inflammatory therapy groups. After dichotomization into impaired or unimpaired cognition, 5 patients (18%) were classified as being cognitively impaired in the group without anti-inflammatory therapy, 7 patients (27%) in the group treated with dexamethasone, and 14 (33%) in the group treated with dexamethasone and tocilizumab (p = 0.361).

Patients who received both dexamethasone and tocilizumab performed significantly worse on the TMT B/A compared to patients without anti-inflammatory therapy (T-score 40.3 ± 13.5 vs 49.1 ± 9.3, p = 0.007). One-way ANOVA on the TMT-A revealed a statistically significant difference in between-group comparisons (p = 0.042), although this was not confirmed when post-hoc analyses with correcting for multiple testing were applied.

**Fig. 3:**
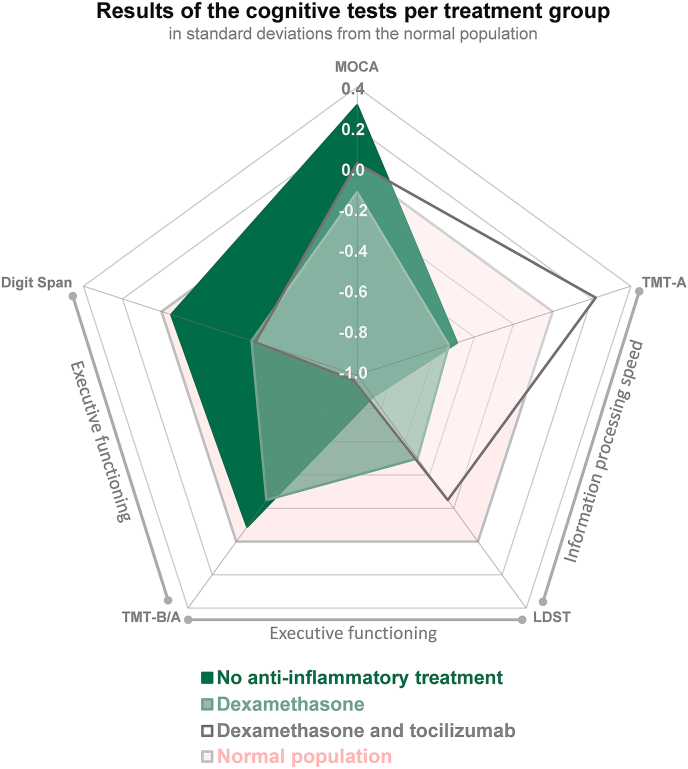
Overview of cognitive test results for each anti-inflammatory treatment group in standard deviations compared to normative population. The y-axis reflects the standard deviation of the treatment groups compared to the normal population. Negative scores correspond with poorer cognition and positive scores with performance above population average. **Abbreviations:** MoCA: Montreal Cognitive Assessment; TMT-A: Trail Making Test A; LDST: Letter Digit Substitution Test; Digit Span: Digit Span subtest from the Wechsler Adult Intelligence Scale - Fourth Edition (WAIS-IV); TMT-B/A: Trail Making Test-B/A ratio, derived from Trail Making Test A and B.

Fig. 3 presents test results in standard deviations compared to normal population, displaying different patterns of cognitive functioning between the treatment groups. On executive functioning tasks, tocilizumab patients scored lowest compared to patients without anti-inflammatory medication, who performed comparable to the normative population. The group treated with only dexamethasone performed intermediate between these two groups. Finally, no statistically significant differences were found in any of the self-report questionnaires between the different treatments.

## 4 Discussion

Cognitive dysfunction represents a serious long-term consequence of critical illness (Wolters et al., 2013) and is especially observed in patients that suffered from infectious diseases (Annane and Sharshar, 2015). In this study, we investigated long-term cognition in survivors of critical illness due to COVID-19. In these severely affected COVID-19 patients, we observed that approximately 3 out of 10 had cognitive impairment six months after ICU discharge, unrelated to baseline demographic characteristics or clinical course. Furthermore, 1 out of 5 patients self-reported severe cognitive complaints, strongly correlating with anxiety and other psychopathological symptoms, but not with objective cognitive test results. In addition, while neuroinflammation is thought to induce cognitive dysfunction, systemic immunosuppressive therapy was not associated with a different occurrence or severity of cognitive dysfunction, apart from a more, not less, pronounced impairment in executive functioning in patients who were treated with the combination of dexamethasone and tocilizumab. Although there seems to be an association between immunosuppressive treatments and worsened executive functioning, causality is difficult to establish due to the temporal bias, number of patients, possible bias due to non-responders (selection bias) or effects of post-ICU rehabilitation care.

Comparing studies that assess neuropsychological function remains troublesome. Long-term cognitive impairment in non-COVID ICU patients ranges between 4 and 62% (Wolters et al., 2013). This large variation is likely due to methodological differences regarding the extensiveness of neuropsychological test batteries (i.e., a single cognitive screening test vs more extensive and sensitive test batteries), follow-up duration and the validity of cut-off values or reference data. A follow-up study of 74 non-COVID ARDS survivors that applied the same cut-off values for neurocognitive impairment as the current study, revealed cognitive deficits in 73% of patients at hospital discharge, still being present in 46% of patients one year later (Hopkins et al., 2005). However, they performed more neuropsychological tests than we did, increasing the probability that patients obtained a low score in at least one of the tests, which could result in a higher percentage of patients classified as cognitively impaired. In a recent cross-sectional cohort study in 196 hospitalized COVID-19 patients (Becker et al., 2021), 13–39% scored cognitively impaired after seven months depending on which of the nine different cognitive domains was assessed. However, no data were provided on disease severity and other important characteristics, such as age, hospital length of stay, whether patients were admitted to the ICU or not, proportion of invasive mechanical ventilation or severity of disease. Lack of such details greatly hampers the use of such data in the daily clinical practice (e.g., for counseling) and also makes it difficult to compare them with our study results.

There is a strong association between occurrence of systemic inflammation and subsequent development of cognitive impairment (Shen et al., 2019). Based on preclinical (Barichello et al., 2019), post-mortal (Westhoff et al., 2019; Schurink et al., 2020) and neuro-imaging (Visser et al., 2002) studies, neuro-inflammation appears to play a relevant role in the development of brain dysfunction following sepsis. Therefore, inhibition of systemic inflammatory mediators might impact the development of cognitive impairment. This study is the first to study the relation between immunomodulatory therapy and cognitive outcomes in patients with COVID-19.

Dexamethasone is a long-acting synthetic corticosteroid that decreases inflammation through suppression of neutrophil migration, dampened production of inflammatory cytokines and reversal of increased capillary permeability (Hill and Spencer-Segal, 2021). Although, the specific mechanisms of action remain unknown, dexamethasone administration is common practice in patients with brain tumors because of its strong effect on reducing cerebral edema and improving neurological symptoms (Dietrich et al., 2011). Whether dexamethasone can protect the brain during critical illness remains unclear. Due to the efflux transporter P-glycoprotein, dexamethasone does not penetrate the blood-brain barrier, although this could be affected if the blood-brain barrier is more permeable during systemic inflammation (Hill and Spencer-Segal, 2021). The reduction of peripherally derived cytokines through dexamethasone is hypothesized to prevent neuroinflammatory activation of glial cells (Hill and Spencer-Segal, 2021). However, although the inhibitory effect of dexamethasone on systemic cytokines was strong in LPS (lipopolysaccharide) stimulated mice, cytokine production within the brain was unaltered (Murray et al., 2011).

Tocilizumab is a recombinant humanized monoclonal antibody IL-6 receptor inhibitor with a poor blood-brain barrier penetration. Elevated IL-6 levels in plasma and cerebrospinal fluid correlated with cognitive decline (Weaver et al., 2002; Sparkman et al., 2006). Up until now, clinical studies on the effect of tocilizumab in neuropsychiatric diseases showed ambiguous results. In patients with rheumatoid arthritis and depression (Figueiredo-Braga et al., 2018), treatment with IL-6 antagonists significantly attenuated depressive symptoms, although this was probably due to the reduction of the systemic auto-immune disease activity itself, while depressive and anxiety symptoms worsened in hematological patients treated with tocilizumab (Knight et al., 2021). A randomized controlled trial of tocilizumab as add-on treatment in schizophrenia did not reveal a difference in behavioral outcomes (Girgis et al., 2018). Our data suggest that tocilizumab might worsen executive functioning. The underlying pathophysiology might be similar as observed in cytokine release syndrome, where tocilizumab treatment may amplify neurotoxicity. It is thought that this is caused by a transient rise of systemic IL-6 levels after tocilizumab administration, due to peripheral IL-6 receptor blockage (Nishimoto et al., 2008). Since tocilizumab does not cross the blood-brain barrier, increased systemic IL-6 concentration could lead to higher levels within the central nervous system, resulting in increased neuroinflammatory responses and neurotoxicity (Lee et al., 2014).

In our study, a higher proportion patients with cognitive impairment had diabetes mellitus and this may be an illustration of previous findings that diabetes has been linked to an increased susceptibility to develop cognitive deficits (Cheng et al., 2012).

Although all neuropsychological test results are adjusted for age, sex and level of education with normative data, it is interesting to note that the majority of cognitively impaired patients in this study received a lower level of education. An explanation could be found in the concept of cognitive reserve: a principle that accounts for the disconnection between the degree of brain damage and clinical outcome, where individuals with less cognitive reserve are more susceptible to the effects of brain disease (Stern, 2009; Stern et al., 2020). Education and IQ are suggested proxies of cognitive reserve. When applied on our study, patients with lower level of education and lower IQ may thus have less cognitive reserve to compensate for the impact of – in this case– ICU admission due to severe COVID-19. Therefore, the same event might have a more pronounced impact in such a patient compared to someone with higher level of education (i.e. more cognitive reserve).

The results of this study also highlight that there is an important clinical difference between *objective* cognitive impairment and *subjective* cognitive complaints, as also reported in previous cognition studies (Jungwirth et al., 2004; Fischer et al., 2010; Blackmon et al., 2022). Patients performing in the cognitively impaired range on neuropsychological tests did not report more subjective cognitive complaints or other psychopathological symptoms than those without cognitive impairment. Psychological affects are found to be associated with poorer subjective cognition, but not with objective cognition (Balash et al., 2013; Byrne et al., 2017; Siciliano et al., 2021). In accordance, in our study, patients with subjective memory impairment reported significantly higher anxiety scores. Furthermore, patients reporting cognitive complaints, were more likely female and appeared to have had *less* critical COVID-19 (i.e., shorter time on ventilator and shorter duration of ICU stay, fewer delirium- and coma-free days). This is in line with a French follow-up study in patients hospitalized for COVID-19, where higher disease severity and ICU admission was also associated with *fewer* cognitive complaints one month after hospital admission (Gouraud et al., 2021).

The main strength of our study is that we performed detailed long-term objective and subjective neuropsychological follow-up assessments in a large number of well described patients who recovered from severe COVID-19. Also, we were able to study the impact of anti-inflammatory therapies on cognition and distress. We carefully adjusted all our cognitive tests and questionnaires for age and level of education and/or sex using extensive normative data, resulting in a more valid assessment of cognitive (dys)function. Limitations of this study include the following. First, we only recruited patients from one hospital. Second, although all COVID-19 ICU survivors were approached for participation, the most fragile – mostly still living in nursing homes – refused more often, possibly resulting in an overestimation of cognitive performance. Third, inherent to the design of the study, there were minor differences in baseline demographic characteristics between the groups that might have affected the observed outcomes. Also, introduction of immunomodulatory treatments prior to ICU admission may have impacted the case-mix of patients that did need intensive care treatment. Finally, although our total cohort is considered relatively large for a post-ICU long-term cognition study, the subgroups were relatively small and therefore limit the statistical power to detect differences on between-group comparisons.

## 5 Conclusion

In a comprehensive sample of patients that survived severe COVID-19, we found that a significant proportion of the patients had cognitive impairment six months post-ICU. Furthermore, administration of IL-6 receptor antagonists and/or dexamethasone did not change the overall incidence of cognitive dysfunction or subjective long-term outcomes.

## Funding

The study was internally funded by a grant of the Radboudumc Center for Infectious Diseases to WFA. WFA was also supported by a research grant from the 10.13039/501100001826Netherlands Organisation for Health Research and Development (ZonMW Clinical Fellowship grant 90715610). The funders of the study had no role in study design, data collection, data analysis, data interpretation, or writing of the report.

### Author contributors

HBD contributed to the overall design, literature search, patient inclusion and data acquisition, data interpretation and manuscript writing. RPCK and WFA contributed to overall design and methodology, data interpretation and supervision of manuscript writing. PP contributed to data interpretation, supervision and critically reviewed the manuscript. BvdB contributed to patient inclusion and data acquisition and critically reviewed the manuscript. All authors had full access to all the data in the study, reviewed and approved the final manuscript text and accept responsibility to submit for publication.

## Declaration of competing interest

The authors declare that they have no conflict of interest related to this study.

## Data Availability

Data will be made available on request.
