# Supplementary material for: Long-term cognitive performance and its relation to anti-inflammatory therapy in a cohort of survivors of severe COVID-19
Source: Brain Behav Immun Health. 2022 Sep 18;25:100513. doi: 10.1016/j.bbih.2022.100513 (PMC9482799; doi:10.1016/j.bbih.2022.100513)
Supplement: Multimedia component 1 [file mmc1.pdf]

Supplemental material to: https://doi.org/10.1016/j.bbih.2022.100513

Supplemental material

Table s1. Neuropsychological test and questionnaire outcomes in patients with and without cognitive impairments or cognitive complaints

	Objective cognition			Subjective cognition		
	**Unimpaired (n=70)**	**Impaired (n=26)**	**p-value**	**Unimpaired (n=74)**	**Impaired (n=18)**	**p-value**
**Neuropsychological tests**						
MoCA raw score, median [IQR]	26 [25-28]	24 [22-26]	**p<0.001**	26 [25-28]	26 [25-26]	p=0.44
MoCA T-score, mean (SD)	52.5 (7.8)	45.2 (10.3)	**p=0.001**	51.4 (9.7)	48.3 (6.0)	p=0.192
TMT-A T- score, mean (SD)	51.3 (11.1)	39.3 (17.6)	**p=0.003**	48.8 (13.4)	45.9 (14.7)	p=0.42
TMT-B T-score, mean (SD)	50.3 (8.8)	29.1 (8.8)	**p<0.001**	45.9 (11.7)	47.0 (9.1)	p=0.71
TMT-B/A T- score, mean (SD)	49.7 (9.3)	31.8 (11.1)	**p<0.001**	45.5 (12.2)	49.0 (9.4)	p=0.26
Digit Span T- score, mean (SD)	49.1 (9.5)	39.4 (11.4)	**p=0.003**	47.3 (11.0)	45.9 (9.5)	p=0.64
LDST T-score, mean (SD)	48.0 (10.0)	37.0 (10.4)	**p<0.001**	45.4 (10.9)	45.5 (12.5)	p=0.97
Overall T-score, mean (SD)	50.1 (5.1)	38.5 (6.5)	**p<0.001**	47.4 (7.4)	46.8 (6.7)	p=0.77
**Weighted test results**						
MoCA, n (%)						
- Unimpaired	66 (94.3)	19 (73.1)	**p=0.001**	66 (89.2)	17 (94.4)	p=0.60
- Below average	4 (5.7)	2 (7.7)		4 (5.4)	1 (5.6)	
- Impaired	0 (0.0)	5 (19.2)		4 (5.4)	0 (0.0)	
TMT-A, n (%)						
- Unimpaired	54 (77.1)	13 (50.0)	**p=0.001**	54 (73.0)	10 (55.6)	p=0.34
- Below average	12 (17.1)	3 (11.5)		11 (14.9)	4 (22.2)	
- Impaired	4 (5.7)	10 (38.5)		9 (12.1)	4 (22.2)	
TMT-B, n (%)						
- Unimpaired	63 (90.0)	3 (11.5)	**p<0.001**	48 (69.6)	15 (83.3)	p=0.51
- Below average	5 (7.1)	3 (11.5)		7 (10.1)	1 (5.6)	
- Impaired	2 (2.9)	20 (76.9)		14 (20.3)	2 (11.1)	
TMT-B/A, n (%)						
- Unimpaired	59 (84.3)	5 (19.2)	**p<0.001**	48 (69.6)	14 (77.8)	p=0.66
- Below average	8 (11.4)	4 (15.4)		7 (10.1)	4 (22.2)	
- Impaired	3 (4.3)	17 (65.4)		14 (20.3)	0 (0.0)	
Digit Span, n (%)						
- Unimpaired	57 (81.4)	11 (42.3)	**p<0.001**	54 (73.0)	13 (72.2)	p=0.99
- Below average	9 (12.9)	2 (7.7)		8 (10.8)	2 (11.1)	
- Impaired	4 (5.7)	13 (50.0)		12 (16.2)	3 (16.7)	
LDST, n (%)						
- Unimpaired	56 (80.0)	9 (34.6)	**p<0.001**	50 (67.6)	14 (77.8)	p=0.30
- Below average	5 (7.1)	4 (15.4)		9 (12.2)	0 (0.0)	
- Impaired	9 (12.9)	13 (50.0)		15 (20.3)	4 (22.2)	
**Self-reporting Questionnaires**						
CFQ total, mean (SD)	32.7 (15.6)	33.8 (14.5)	p=0.76	27.3 (10.8)	56.6 (8.8)	**p<0.001**
Clinical Frailty Scale, median [IQR]	3 [2-4]	4 [3-4]	p=0.12	3 [2-4]	4 [3-4]	p=0.18
HADS total, median [IQR]	7 [4-15]	9 [5-17]	p=0.24	7 [4-14]	15 [8-21]	**p=0.002**
HADS anxiety, median [IQR]	4 [2-8]	4 [3-10]	p=0.27	3 [2-6]	8 [6-11]	**p<0.001**
HADS depression, median [IQR]	3 [2-7]	5 [3-7]	p=0.12	3 [2-7]	5 [3-12]	p=0.090
SF-12 physical T-score, median [IQR]	38.0 [30.6-48.1]	37.7 [31.6-41.7]	p=0.76	39.3 [32.1-48.1]	32.9 [23.0-39.4]	**p=0.026**
SF-12 mental T-score, median [IQR]	52.7 [43.6-57.9]	54.9 [40.6-56.6]	p=0.29	53.8 [46.5-59.1]	43.0 [37.8-51.3]	**p=0.004**
BSI (GSI) T-score, median [IQR]	48.0 [36.4-59.8]	54.9 [39.9-62.0]	p=0.13	47.1 [36.4-56.8]	63.7 [58.2-68.9]	**p<0.001**
**Dichotomized cognitive outcome**						
Objective cognitive impairment, n(%)	0 (0.0)	24 (100.0)	NA	21 (28.4)	3 (16.7)	p=0.31
Subjective cognitive impairment, n(%)	15 (22.1)	3 (12.5)	p=0.31	0 (0.0)	18 (100.0)	NA

Abbreviations: MoCA, Montreal Cognitive Assessment; TMT, Trail Making Test; Digit Span, Wechsler Adult Intelligence Scale-IV Digit Span test; LDST, Letter Digit Substitution Test. CFQ, Cognitive Failure Questionnaire; HADS, Hospital Anxiety and Depression Scale; SF-12, Short Form Health Survey-12; BSI, Brief Symptom Inventory; GSI, Global Severity Index. All individual test results were corrected for age, education, and sex using normative data of normal Dutch control population. **Weighted test results:** *unimpaired*: T-score >40 (> -1 SD); *below average*: T-score 35-40 (-1.5 to -1 SD); *impaired*: T-score <40 (< -1.5 SD). **Dichotomization**: Patients were categorized as *objectively* cognitively impaired if they had a weighted score of *impaired* at two or more tests. Patients were categorized as *subjectively* cognitively impaired when CFQ scores were 44 or higher.
